# Industrial wastewater as raw material for exopolysaccharide production by *Rhizobium leguminosarum*

**DOI:** 10.1590/S1517-838246220140153

**Published:** 2015-06-01

**Authors:** Mohamed Sellami, Tomasz Oszako, Nabil Miled, Faouzi Ben Rebah

**Affiliations:** 1 Laboratoire de Biochimie et de Génie Enzymatique des Lipases, Ecole Nationale d'Ingénieurs de Sfax, Université de Sfax, Sfax, Tunisia, Laboratoire de Biochimie et de Génie Enzymatique des Lipases, Ecole Nationale d'Ingénieurs de Sfax, Université de Sfax, Sfax, Tunisia.; 2 Instytut Badawczy Lesnictwa, Forest Research Institute, Sekocin Stary, ulica Raszyn, Poland, Instytut Badawczy Lesnictwa, Forest Research Institute, Sekocin Stary, ulica Raszyn, Poland.; 3 King Khaled University, King Khalid University, Community College at Khamis Mushait, Khamis Mushait, Saudi Arabia, King Khalid University, Community College at Khamis Mushait, Khamis Mushait, Saudi Arabia.

**Keywords:** industrial wastewater, biopolymer, exopolysaccharide, Rhizobium

## Abstract

The objective of this study was to evaluate the exopolysaccharide (EPS) production by *Rhizobium leguminosarum* cultivated in wastewater generated by oil companies (WWOC1 and WWOC2) and fish processing industry (WWFP). The results obtained in Erlenmeyer flasks indicated that the rhizobial strain grew well in industrial wastewater. Generally, wastewater composition affected the growth and the EPS production. WWFP allowed good bacterial growth similar to that obtained with the standard medium (YMB). During growth, various quantities of EPS were produced and yields varied depending on the media. Growing in YMB, EPS production did not exceed 9.7 g/L obtained after 72 h of growth. In wastewater, the maximum EPS value reached 11.1 g/L obtained with the fish processing wastewater, after 72 h of growth. The use of a mixture of the oil company wastewater (WWOC2) and the fish processing wastewater (WWFP) as culture medium affected not only the rhizobial strain growth, but also EPS production. The highest EPS (42.4 g/L, after 96 h of culture) was obtained using a ratio of WWFP and WWOC2 of 50:50 (v:v). Therefore, this work shows the ability of *Rhizobium leguminosarum*, growing in industrial wastewater as new economic medium, to produce EPS. This biopolymer could be applied in enormous biotechnological areas.

## Introduction

In recent years, scientists are interested in natural polymers generally obtained from plants (Farooq *et al.*, 2014), animals (Kumar *et al.*, 2000) and microorganisms (Mukherjee *et al.*, 2010). For sustainable and economical production of bioactive polysaccharides at industrial scale, rather than plants and algae, microbial sources are preferred since they enable fast and high yielding production processes under fully controlled fermentation conditions (Villano *et al.*, 2013; Wang *et al.*, 2012). Among the biopolymers, polyhydroxybutyrate (PHB) and exopolysaccharides (EPS) stand out because of its applications, mainly in biodegradable plastic production and in food industry, respectively. PHB is a microbial polyester stored in cells in the form of granules (Tavernier *et al.*, 2008). However, EPS is excreted in the growth media (Kumari *et al.*, 2009). Being obtained from renewable sources, they bear specific features such as biocompatibility, biodegradability, non-toxicity, wide availability and low cost. In this context, the capacity of Rhizobia to produce EPS and PHB was evaluated in many studies. Rhizobial EPS is a very important for proper biofilm formation both on abiotic surfaces and on roots of the host plants (Xie *et al.*, 2012); is involved in the *Rhizobium*-Legume symbiosis (Fraysse *et al.*, 2003). EPS Biosynthesis in rhizobia is a complex process regulated at both transcriptional and post-transcriptional levels and controlled by several nutrients and environmental parameters (Janczarek, 2011). Interestingly, EPS have been fully explored because of their enormous applications in medicine, agriculture and food industries such as emulsifiers, stabilizers, binders, gelling agents, coagulating agents, flocculating agents, film-forming substances, lubricants, thickening agents, immunostimulating agents and antitumor agents (Donot *et al.*, 2012; Freitas *et al.*, 2011; Nicolaus *et al.*, 2010). Generally, the nature and the proportion of polymer produced by Rhizobia are controlled by several factors, such as the composition of the culture medium, fermentation conditions (pH, temperature, oxygen concentration) and the carbon source used during culture (Duta *et al.*, 2006). Moreover, biopolymer utilization depends on the production cost which is mainly related to the raw material used as growth medium. In order to reduce the production cost, the use of cheaper carbon source is needed. In this perspective, much effort has been spent in optimizing the PHB production using pure substrates and pure cultures. More recently, sludge generated by industrial and municipal wastewater-treatment process, a worldly recyclable waste, has shown good potential to be used as a growth medium and as a carrier (dehydrated sludge) for rhizobia-based inoculant production (Ben Rebah *et al.*, 2001; 2002; 2009). However, no studies have examined the feasibility of utilizing rhizobial strains growing in industrial wastewater to produce EPS. Therefore, this work aims at studying the capability of producing EPS by *Rhizobium leguminosarum* growing in industrial wastewater, which is considered as abundant and inexpensive substrate. This approach can lower the cost of EPS production and, simultaneously reduce environmental problems associated with industrial wastewater treatment.

## Materials and Methods

### Wastewater sampling and characterization

Two types of wastewater from oil companies (WWOC1 and WWOC2) and a fish processing wastewater (WWFP) were collected and stored at 4 °C until their use. WWOC1 and WWOC2 represented the wastewater generated during the cleaning process of the oil drilling equipments. However, the WWFP is generated during operations such as cleaning, cooling, thawing, etc. The pH was measured with a pH meter (Orion model 420A). Total solids (TS), lipids, total Kjeldahl nitrogen (TKN), biochemical oxygen demand (BOD_5_) and chemical oxygen demand (COD) were determined according to the Standard Methods (APHA, 1992).

### Micro-organism

*Rhizobium leguminosarum* ATCC 10004 was used in this study. Culture was maintained at 4 °C on mannitol agar slants.

### Inoculum preparation

The inoculum for the experiments was prepared by growing rhizobial strain in 250 mL Erlenmeyer flasks containing 25 mL of the sterilised standard medium (YMB: Yeast Mannitol Broth). The flask was incubated at 30 °C for 48 h on a rotary shaker at 200 rpm. The standard medium contained the following constituents (in grams per liter): K_2_HPO_4_, 0.5; MgSO_4_ 7H_2_O, 0.2; NaCl, 0.1; yeast extract, 1 and mannitol, 10.

### Growth experiments

The initial pH of the wastewater samples was adjusted to 7.0 using either NaOH or H_2_SO_4_. The samples were sterilised at 121 °C for 20 min. Growth experiments were carried out in 500 mL Erlenmyer flasks each containing 100 mL of the sterile medium (wastewater or standard medium). Flasks were inoculated with 4% (v/v) of the inoculum. Conditions used in the experiments were the same as those used to prepare the inoculum. The samples (1.5 mL) were drawn at regular intervals. The cell count was performed on agar plates using YMA (Yeast Mannitol Agar) with Congo red (0.25%) after appropriate serial dilution of 0.5 mL samples with saline solution (NaCl 0.85%). The exopolysaccharide production was determined from 1 mL samples taken during rhizobial growth. First, the bacteria were removed from the medium by centrifugation (30 min at 3500 × *g*), the supernatants were collected and the EPS were precipitated by two volumes of chilled acetone. The crude polysaccharide developed was collected by centrifugation at 3500 × *g* for 30 min. Then, suspended in distilled water (1 mL), reprecipitated with acetone alternately, transferred onto a filter paper and weighed after overnight drying at 105 °C (Kumari *et al.*, 2009). All experiments were conducted in triplicate.

## Results

The characterization of industrial wastewater samples are presented in Table 1. The composition of raw materials, provided by different industries varied depending on their origins. The wastewater generated by the fish processing industry (WWFP) had higher concentrations of total solids (50.77 g/L), COD (4.77 g/L), BOD_5_ (4 g/L), total Kjeldahl nitrogen (1.05 g/L) and lipids (148.26 g/L) than those of the samples provided by oil companies (WWOC1 and WWOC2). However, the highest C/N ratio was observed in the case of WWOC2 (C/N (COD/TKN) = 39.28).

**Table 1:** Characterisation of industrial wastewater used in the experiment.

Parameters	WWOC1	WWOC2	WWFP
pH	6.56	11.99	6.54
Total solids (g/L)	43.25	17.46	50.77
Lipids (g/L)	5.25	0.75	148.26
COD (g d'O_2_/L)	2.32	1.10	4.77
BOD_5_ (g d'O_2_/L)	0.10	0.40	4.00
TKN (g/L)	0.28	0.028	1.05
C/N (COD/TKN)	8.28	39.28	4.54

WWOC1: wastewater from the oil company 1; WWOC2: wastewater from the oil company 2; WWFP : wastewater from the fish processing industry.

### Industrial wastewater as medium for rhizobial biomass and EPS production

**Figure 1:**
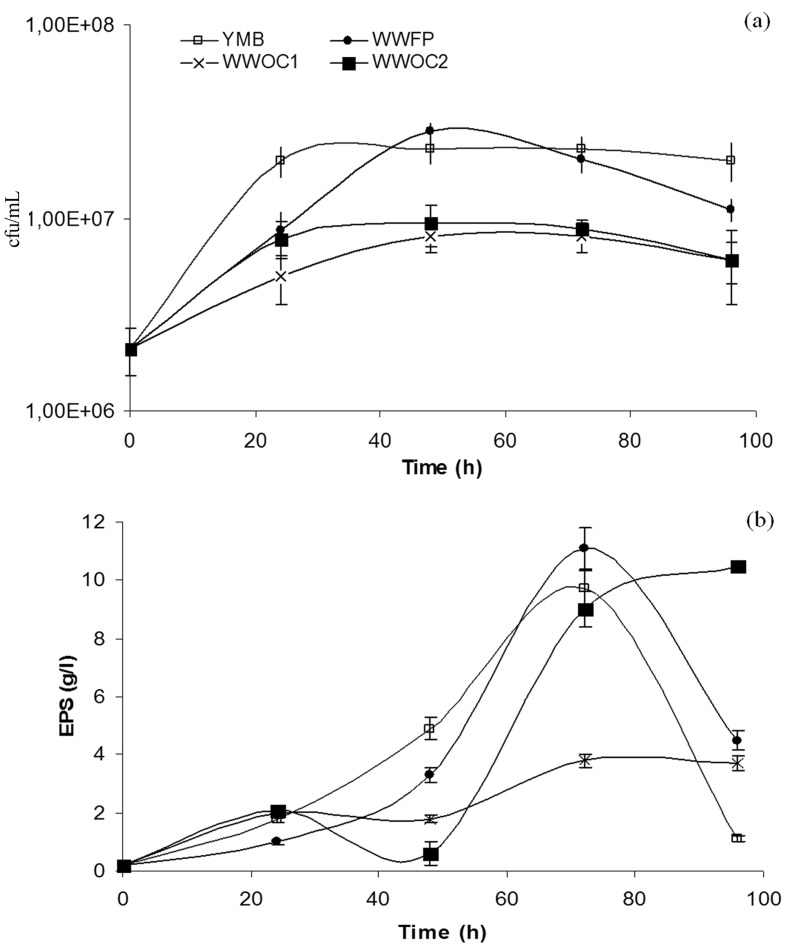
Growth (a) and EPS production (b) of *R. leguminosarum* grown in industrial wastewater from various origins.

A fast-growing strain (*Rhizobium legumunosarium*) was grown in industrial wastewater (WWOC1, WWOC2 and WWFP) and in YMB (as a control). According to Figure 1a, all wastewater samples sustained the bacterial growth. The WWFP gave a maximum cell concentration (2.80 × 10^7^ cfu/mL, obtained after 48 h of growth) comparable to that obtained in YMB medium (2.27 × 10^7^ cfu /mL, obtained after 48 h of growth). For the other two effluents, maximum cell concentrations were respectively 0.8 × 10^7^ cfu /mL (for WWOC1, after 48 h of culture) and 0.94 × 10^7^ cfu /mL (for WWOC2 after 48 h of culture) (Table 2).

**Table 2:** Results of *R. leguminosarum* growth in standard medium and in industrial wastewater; mean ± SD.

	Growth		EPS production	
		
	X_max_	T_Xmax_	EPS_max_	T_EPSmax_
YMB	2.27 ± 0.32 × 10^7^	72	9.70 ± 0.60	72
WWFP	2.80 ± 0.28 × 10^7^	48	11.10 ± 0.71	72
WWOC1	0.80 ± 0.14 × 10^7^	48	3.80 ± 0.23	72
WWOC2	0.94 ± 0.22 × 10^7^	48	10.50 ± 0.11	96

YMB : Yeast Mannitol Broth; WWOC1: wastewater from the oil company 1; WWOC2: wastewater from the oil company 2; WWFP : wastewater from the fish processing industry; X_max_ : maximum cell count (cfu/mL); T_Xmax_ : time of the obtained X_max_ (h); EPS_max_: maximum EPS production (g/L); T_EPSmax_ : time of the obtained EPS_max_ (h).

The EPS production started simultaneously with the growth, but attained its maximum in the stationary phase (Figure 1b). Depending on the growth media, a lag phase was observed and the EPS amount do not exceeded 4.9 g/L during the first 48 h of culture (obtained in the presence of YMB medium).

After 48 h of culture, considerable EPS enhancement was observed for YMB, WWFP and WWOC2 with maximum values of 9.7 g/L (after 72 h), 11.1 g/L (after 72 h) and 10.5 g/L (after 96 h), respectively. However, for the WWOC1, the EPS yields remained at lower level and do not exceeded the maximum value of 3.79 g/L obtained after 72 h of culture (Table 2). After reaching the maximum, the EPS amount declined. The EPS decline was significant in the case of YMB and WWFP media. However, in the case of WWOC1 and WWOC2, the EPS amount remained almost constant.

**Figure 2:**
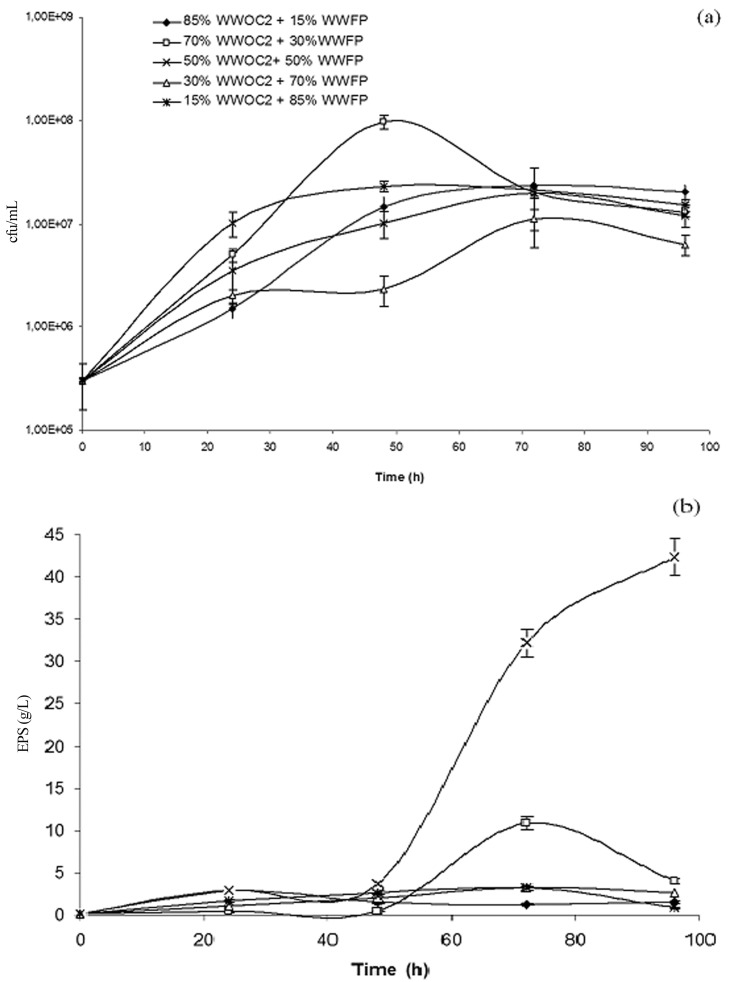
Growth (a) and EPS production (b) of *R. leguminosarum* grown in wastewater samples from oil company (WWOC2) and fish processing industry (WWFP) mixed at different proportions.

The use of WWOC2 and WWFP mixed at different proportions influenced the rhizobial growth and EPS production (Figure 2, Table 3). The maximum cell count was obtained using a ratio of WWOC2 and WWFP of 70:30 (v:v). The highest value of 9.70 × 10^7^ cfu/mL was reached after 48 h of culture. As far as the EPS production is concerned, a lag phase was observed for all samples and the mixture containing 50% of both WWOC2 and WWFP increased nearly fourfold the EPS as compared with YMB, WWOC2 and WWFP tested separately. However, this value of 42.4 g/L was obtained at 96 h with a cell count of 2.30 × 10^7^ cfu/mL (Table 3). Except, for the mixture 50:50 (v:v.), a decrease in EPS production was also noticed.

**Table 3:** Results of *R. leguminosarum* growth in WWCO2 and WWFP mixed at different proportions; mean ± SD.

Growth media	Growth		EPS production	
		
	X_max_	T_Xmax_	EPS_max_	T_EPSmax_
100% WWOC2	0.94 ± 0.22 × 10^7^	48	10.50 ± 0.11	96
100% WWFP	2.80 ± 0.28 × 10^7^	48	11.10 ± 0.71	72
85% WWCO2 + 15% WWFP	2.35 ± 0.43 × 10^7^	72	3.00 ± 0.24	24
70% WWCO2 + 30% WWFP	9.70 ± 1.40 × 10^7^	48	10.90 ± 0.78	72
50% WWCO2 + 50% WWFP	2.30 ± 0.28 × 10^7^	48	42.40 ± 2.16	96
30% WWCO2 + 70% WWFP	1.12 ± 0.25 × 10^7^	72	3.30 ± 0.16	72
15% WWCO2 + 85% WWFP	2.00 ± 1.41 × 10^7^	72	3.20 ± 0.24	72

WWOC2: wastewater from the oil company 2; WWFP: wastewater from the fish processing industry; X_max_ : maximum cell count (cfu/mL); T_Xmax_ : time of the obtained X_max_ (h); EPS_max_: maximum EPS production (g/L); T_EPSmax_ : time of the obtained EPS_max_ (h).

## Discussion

Microbial exopolysaccharides have been fully explored because of their original structure, their chemical and rheological properties in solution which confer them good industrial applications in various industries (cosmetic, food-processing, pharmaceutical industry, etc.) (Tavernier *et al.*, 2008). Therefore, many strategies were used for large scale production of EPS using selected bacteria and economic growth media (Kumar *et al.*, 2001).

Various rhizobial strains are known to produce large amounts of EPS both in the rhizosphere and when grown in pure culture (Datta and Basu, 1999; Noel and Schaechter, 2009). As a matter of fact, we tried to produce EPS by growing rhizobial strain in industrial wastewater. This can lower the cost of growth media for EPS production and reduce the wastewater handling and disposal. Rhizobium was able to grow using nutrient contained in wastewater. Growth parameters were affected by the nature of the used medium. These results confirmed those reported by Ben Rebah *et al.* (2001; 2002; 2009) who have used sludge generated by industrial and municipal wastewater-treatment process as a growth medium for rhizobia-based inoculants production. Bekele *et al.* (2013) have studied effects of different carbon and nitrogen sources on the growth of *R. leguminosarum* and have shown that using molasses and baker's yeast as carbon and nitrogen sources, respectively exhibited a high growth of the Rhizobium as compared to the standard medium.

The effects of growth media on biomass formation and EPS production by *R. legumenisarium* were studied during 96 h of culture. Both growth and EPS production started simultaneously, but the EPS production was at its maximum in the stationary phase of the growth after 48 h. For all samples, after the lag phase, EPS biosynthesis starts already in logarithmic growth phase and continues in stationary phase reaching a maximum. In some cases, a decline in the amount of EPS was observed. Generally, the lag phase was observed independently of rhizobial strains and culture media (carbon and nitrogen source). Its origin may be related to the synthesis of the EPS precursor which is probably low (Tavernier *et al.*, 1997). The highest EPS production rate (11 g/L) was obtained in fish-processing wastewater (WWFP) which can be related to its higher carbon content (COD = 4.77 g/L). The lower EPS amount (maximum of 3.79 g/L) on WWOC1 may be explained by the insufficient enzymatic activities on the carbon source present in this wastewater. This explanation was reported by Navarini *et al.* (1997) while growing *Rhizobium hedysari* HCNT1 on lactose and maltose (insufficient galactosidase and maltase activities were suggested). Also, the nitrogen source may influence EPS production rate, since the nature of the nitrogen source is not known. Thus, is very important to indicate that nutrient limitations of nitrogen, phosphorus and sulphur have all a stimulating effect on polysaccharide production (Weisany *et al.*, 2013). However, multiproductive patterns of polysaccharides production have been reported in many studies (Sayyed *et al.*, 2011). EPS yields seem to be related to various factors (strains, carbon, nitrogen, etc.) (Duta *et al.*, 2006; Duta *et al.*, 2004). For example, it was reported that *Rhizobium hedysari* HCNT1 reached a highest EPS yield of 9 g/L in presence of mannitol and lysine (Navarini *et al.*, 1997). Some nitrogen sources such as ammonium sulphate and yeast extract may allow optimum EPS yields and other source such as urea may inhibit both growth and EPS production (Sayyed *et al.*, 2011). According to Breedveld *et al.* (1993), NH_4_^+^ is the preferred nitrogen source for several rhizobial species at constant pH. Moreover, C/N ratio may have a role in EPS production and more EPS have been reported with higher C/N value (Sayyed *et al.*, 2011). This underlines the importance of nitrogen and carbon for microbial growth and synthesis of biopolymer. In our study, is very difficult to conclude on the nature of the compounds in wastewater which could affect positively or negatively the rhizoibal biomass and EPS production. Also, we cannot take on consideration the C/N ratio because of the fact that the amount of the available nutrient for the strain was unknown in wastewater. Therefore, it is very important to determine the nature of carbon and nitrogen in wastewater (Nitrate, NH_4_^+^, NTK-N).

Regarding the decline in the amount of EPS, it is may be due to the mobilization of EPS by the organism itself probably under the influence of EPS hydrolyse and the exhaustion of assimilated carbon source (Sayyed *et al.*, 2011). It is well known that fast growing bacteria accumulate organic acids in the standard medium (in the presence of mannitol, glucose, galactose or NH_4_Cl), and the acidification resulted in a repression of polysaccharide synthesis (Courtois *et al.*, 1979). In the case of WWOC1, the absence of EPS decline phase may be explained by its high buffering capacity that could neutralises the produced acids and reducing, consequently, the EPS repression.

WWFP promoted the microbial growth and EPS production better than the other assayed media (YMB, WWOC1 and WWOC2). These results could be explained by the superior availability and biodegradability of nutrients in WWFP and its high contents of amino acids resulted from the fish processing. Interestingly, the WWFP amendments to WWOC2 affected growth and EPS production. The addition of the adequate proportion of the WWFP (50%) may provide, at an acceptable level, various growth factors (amino acids, vitamins, etc.) missing in the WWOC2. The higher level of EPS (42.4 mg/L) produced in mixture of industrial wastewaters (50% WWFP + 50% WWOC2) could be useful for the industry. Hence, the potential of growing Rhizobium in industrial wastewater from food or petrochemical industries may lower the cost of biopolymer production. It seems that both biomass and EPS production were controlled by the composition of growth media. An adequate balance of nutrients and specific factors would ensure higher EPS production. At present, it is difficult to predict the essential growth stimulating factors contained in the wastewater. Therefore, it is very important to determine the nature of carbon and nitrogen compounds contained in each industrial wastewater.

## Conclusion

In this study, the feasibility of using industrial wastewater, generated by oil companies and fish processing industry, as base media for EPS production by *Rhizobium leguminosarum* was conducted. This work can provide alternative substrates for biopolymer production and may help to reduce pollution problems related to wastewater treatment. However, more investigations are needed to examine factors affecting EPS production from wastewater, which are considered as abundant and inexpensive substrates.
